# COVID−19 hospitalization increases the risk of developing glioblastoma: a bidirectional Mendelian-randomization study

**DOI:** 10.3389/fonc.2023.1185466

**Published:** 2023-08-21

**Authors:** Jiajun Dong, Shengnan Wang, Haoqun Xie, Yanhao Mou, Hao Zhu, Yilong Peng, Jianxin Xi, Minggu Zhong, Zhengyuan Xie, Zongyuan Jiang, Kang Wang, Hongyu Chen, Wenzhuo Yang, Mingqin Zhu, Yufeng Wen, Yi Wu

**Affiliations:** ^1^ Department of Neurosurgery, Jiangmen Central Hospital, Jiangmen, China; ^2^ Department of Neurology, The First Hospital of Jilin University, Changchun, China; ^3^ Department of Neurosurgery, Cancer Hospital of Sun Yat sen University, Guangzhou, China; ^4^ Department of Neurosurgery, State Key Laboratory of Oncology in South China, Collaborative Innovation Center for Cancer Medicine, Sun Yat-sen University Cancer Center, Guangzhou, China; ^5^ Department of Hepatology, The First Hospital of Jilin University, Changchun, China; ^6^ Clinical College, Jilin University, Changchun, China; ^7^ Department of Bioengineering, University of Texas at Arlington, Arlington, TX, United States

**Keywords:** Mendelian randomization, COVID-19, SARS-CoV-2, glioblastoma, genome-wide association study

## Abstract

**Background:**

As a result of the COVID-19 pandemic, patients with glioblastoma (GBM) are considered a highly vulnerable population. Despite this, the extent of the causative relationship between GBM and COVID-19 infection is uncertain.

**Methods:**

Genetic instruments for SARS-CoV-2 infection (38,984 cases and 1,644,784 control individuals), COVID-19 hospitalization (8,316 cases and 1,549,095 control individuals), and COVID-19 severity (4,792 cases and 1,054,664 control individuals) were obtained from a genome-wide association study (GWAS) from European populations. A total of 6,183 GBM cases and 18,169 controls from GWAS were enrolled in our study. Their associations were evaluated by applying Mendelian randomization (MR) including IVW meta-analysis, MR-Egger regression, and weighted-median analysis. To make the conclusions more robust and reliable, sensitivity analyses were performed.

**Results:**

Our results showed that genetically predicted COVID−19 hospitalization increases the risk of GBM (OR = 1.202, 95% CI = 1.035–1.395, p = 0.016). In addition, no increased risk of SARS-CoV-2 infection, COVID-19 hospitalization and severity were observed in patients with any type of genetically predicted GBM.

**Conclusion:**

Our MR study indicated for the first time that genetically predicted COVID−19 hospitalization was demonstrated as a risk factor for the development of GBM.

## 1 Introduction

In Coronavirus disease 2019 (COVID-19), the severe acute respiratory syndrome Coronavirus 2 (SARS-CoV-2) is responsible for the illness (1). Since January 1, 2023, more than 661 million people have died from COVID-19, which has caused the tragic loss of over 6 million lives. Coronavirus infections may cause acute cardiac or kidney injury, acute respiratory distress syndrome, shock, secondary infection, cancer, and high mortality risk (2). According to epidemiological studies, cancer is an independent adverse prognostic factor for COVID-19 outcomes, including admission to the intensive care unit and invasive ventilation (3–5). It is especially true for patients suffering from glioblastoma (GBM), which is one of the most aggressive and common types of primary brain tumor. Several factors make GBM patients one of the most fragile and vulnerable cancer populations. Firstly, GBM patients tend to be old age and have multiple age-related comorbidities. Additionally, their large use of steroid medications further increases immunosuppression. Furthermore, there is an increased risk of tumor and/or chemotherapy-related thromboembolic events due to the patient’s loss of autonomy. These result in a greater susceptibility to infection (6).

Besides, the role of COVID-19 in GBM was also a topic of interest. Angiotensin-converting enzyme 2 (ACE2) receptor molecules on the cell membrane interact with the viral spike (S) glycoprotein to allow viral entry (7). According to several studies, the viral S protein binds to the VEGFR (Vascular Endothelial Growth Factor Receptor) and the EGFR (Epidermal Growth Factor Receptor) more frequently in GBM cells than in other types of cancer cells, contributing to their development (8). It has been suggested that COVID-19 infections are associated with a unique brain predisposition to thrombosis caused by cytokine storms (9), which is correlated with faster GBM development. Poor prognosis is associated with tumor thrombus in GBM (10).

Several studies have shown a close relationship between GBM and COVID-19 susceptibility and severity, and traditional observational studies are biased by unmeasured confounding factors, making it difficult to speculate on their causal relationship (6, 11–13). However, observational studies are susceptible to unmeasured confounding or reverse causality. Single-nucleotide polymorphisms (SNPs) are used as instrumental variables (IVs) in Mendelian randomization (MR) studies to examine causal relationships between risk factors and outcomes (14, 15). We used the MR method to evaluate the causal associations between GBM and COVID-19 outcomes, given the limitations of the current research.

## 2 Methods

### 2.1 Study design

**Figure 1:**
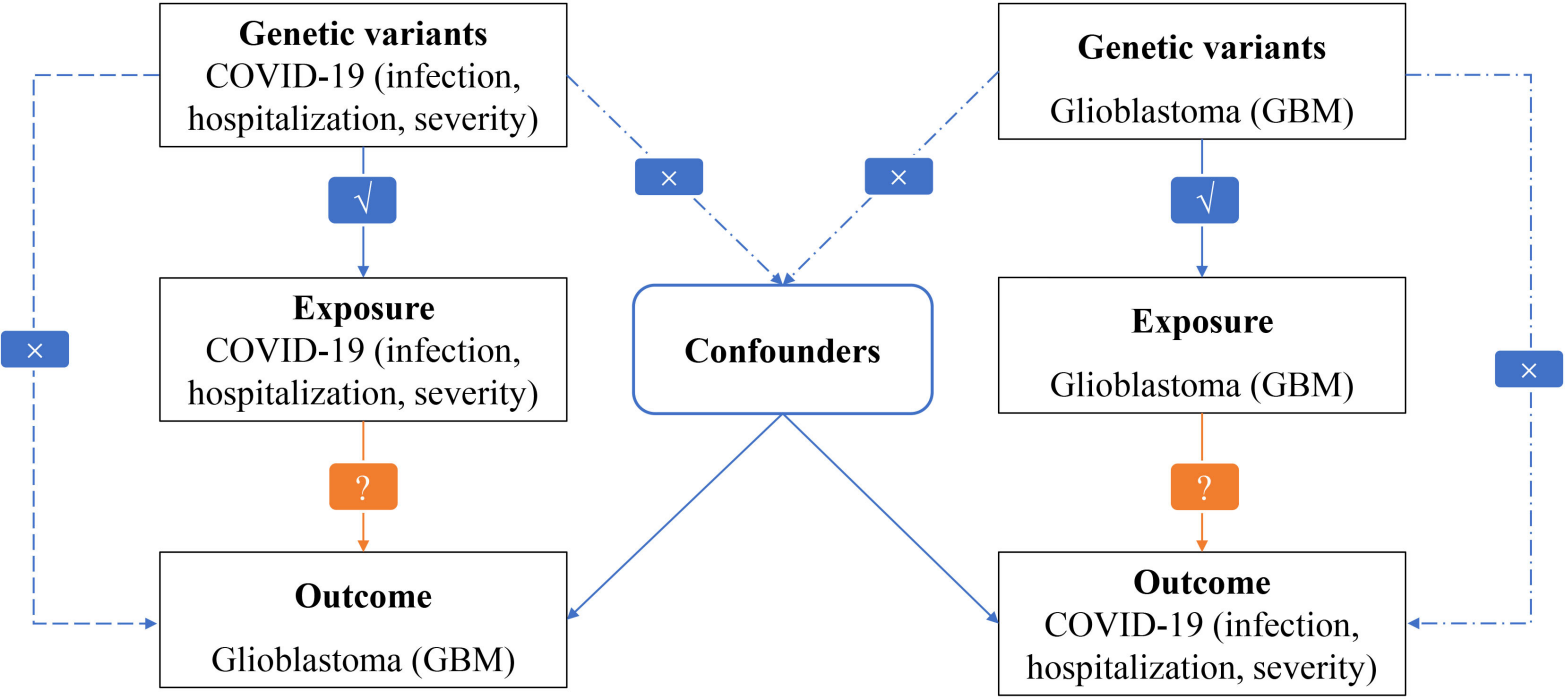
Flowchart of MR analysis in this study.

The overall design of our MR study exploring the causal relationships between GBM and COVID-19 outcomes can be seen in **Figure 1**. The study was conducted on a bidirectional two-sample univariable design. To estimate the causal effects of GBM on COVID-19, a genetically predicted GBM risk is used as an exposure and COVID-19 severity, hospitalization, or susceptibility is used as an outcome (16, 17). Based on genetically predicted COVID-19 severity, hospitalization, and susceptibility risks, we estimate the causal effects of COVID-19 on GBM. MR analysis is based on three critical assumptions: (i) There is a strong association between exposure and IVs; (ii) Confounders should not affect IVs due to exposure and outcome; and (iii) Only exposure mediates IV-outcome associations.

### 2.2 Data sources and instruments selection

We obtained summary-level GWAS data for the datasets of genetically predicted COVID-19 risk (38,984 cases and 1,644,784 control individuals for SARS-CoV-2 infection, 8,316 cases and 1,549,095 control individuals for COVID-19 hospitalization, and 4,792 cases and 1,054,664 control individuals for COVID-19 severity) (18). Participants in this GWAS were from the COVID-19 Host Genetics Initiative, which is publicly available (19). Those with very severe respiratory confirmed COVID-19 were defined as those hospitalized for lab confirmed SARS-CoV-2 infection and dying or requiring respiratory support (20). GBM is defined as glioblastoma (World Health Organization grade IV). GBM genotyping data were derived from a meta-analysis of GWAS, which included 6183 cases and 18169 controls (21). We provided comprehensive details of the genotyping and quality control of COVID-19 and GBM’s GWASs (**Supplementary Table 1**). We used 1000-Genome imputed GWAS data from the European GWAS (22). The mean sample size was larger for COVID-19 and GBM traits, so the genome-wide significance threshold (p < 5×10^–9^) was used to avoid selecting false positive instruments. In the European 1000G reference panel, SNPs with the lowest p-values were retained as independent SNPs after pruning all SNPs in linkage disequilibrium (LD; r^2^ < 0.0001) (23).. We cross-checked the Phenoscanner database (http://www.phenoscanner.medschl.cam.ac.uk/) to identify SNPs associated with the exposure that could potentially be linked to confounding variables or outcomes. We calculated the F-statistic for each SNP to evaluate the strength of the IVs (24, 25). Instruments with an F-statistic below 10 are considered weak (26). **Supplementary Table 2** showed the characteristics of all the SNPs included in our analysis.

### 2.3 Statistical analysis

Several MR analytical methods were used to assess the causal effects and evaluate the potential pleiotropic effects of genetic variants. The main analysis was conducted using inverse-variance weighted (IVW) regression, which assumes no directional pleiotropic effects of individual instrumental variables (27). Weighted median and MR-Egger regression methods were used in complementary analyses (28). Additionally, we conducted sensitivity analyses using the Mendelian Randomization Pleiotropy RESidual Sum and Outlier (MR-PRESSO) global test, MR-Egger intercept, and leave-one-out sensitivity analysis. The sensitivity analysis was tested using a leave-one-out sensitivity analysis by removing each SNP from the analysis and re-estimating its causal effect (29). The horizontal pleiotropy of IVs was assessed by MR-PRESSO and MR-Egger intercept methods (p< 0.05 was considered significant) (30). SNPs with outliers are investigated in the MR-PRESSO global test (31). To test heterogeneity, IVW, and MR-Egger in Cochran’s Q statistic were used (p< 0.05 was considered significant) (32, 33). If the results of the IVW method are significant (p< 0.05), and no pleiotropy and heterogeneity were found, even if the results of other methods were not significant, as long as the beta values of other methods were in the same direction, they could be considered as positive results (34). In R (version 4.2.1), MRPRESSO (version 1.0) and TwoSampleMR (version 0.5.6) were used for the analyses.

## 3 Results

**Figure 2:**
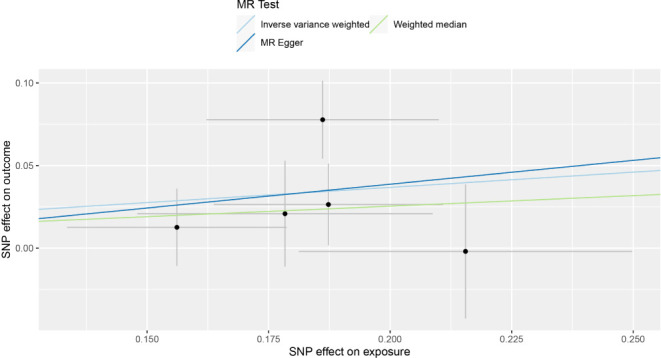
Individual estimates about the putative causal effect of COVID‐19 hospitalization on the risk of GBM.

**Figure 3:**
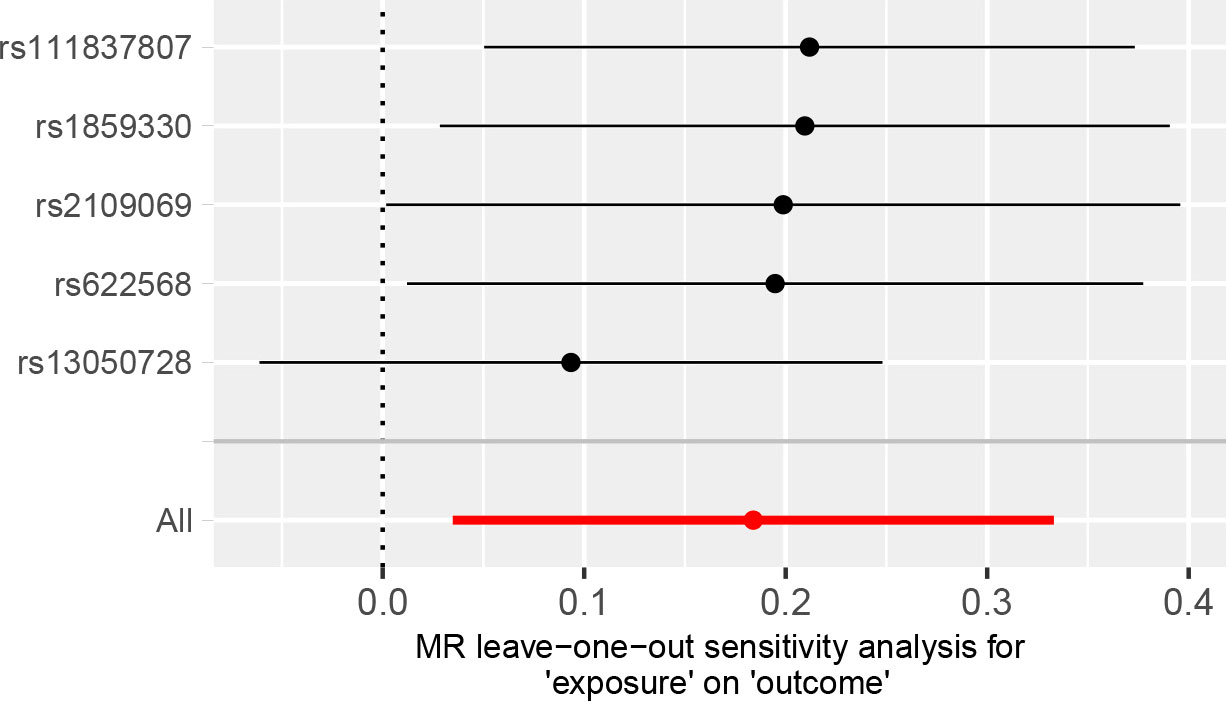
MR leave‐one‐out sensitivity analysis for the effect of COVID‐19 hospitalization SNPs on the risk of GBM.

A comparison of MR estimates obtained from different methods of determining whether COVID-19 causes GBM is presented in **Table 1**. The IVW analysis revealed that the genetically determined COVID-19 hospitalized patients were at higher risk of developing GBM than the general population (OR = 1.202, 95% CI = 1.035–1.395, p = 0.016) (**Figure 2**). According to the MR-Egger intercept, there was no evidence of horizontal pleiotropy (p=0.918). Additionally, there was no obvious heterogeneity (all p-values were >0.05). In leave-one-out analysis, the effect of COVID‐19 SNPs on GBM was robust. For sensitivity analysis, leave-one-out studies were used and showed no influence (**Figure 3**). Furthermore, no significant association was found between severe COVID-19, SARS-CoV-2 infection, and the risk of GBM.

**Table 1:** Association of COVID‐19 genetic IVs with GBM GWAS.

Exposure	Outcome	Method	Number of snps	Beta	P	OR (95%CI)	P for heterogeneity test	P for MR-Egger intercept
SARS-CoV-2 infection	GBM	IVW	4	-0.016	0.934	0.984 (0.676-1.434)	0.184	
		MR Egger	4	-0.553	0.418	0.575 (0.197-1.677)	0.210	0.404
		Weighted median	4	-0.096	0.610	0.908 (0.627-1.314)		
COVID-19 severity		IVW	6	0.089	0.125	1.093 (0.976-1.225)	0.194	
		MR Egger	6	-0.384	0.139	0.681 (0.453-1.024)	0.750	0.080
		Weighted median	6	0.082	0.181	1.086 (0.962-1.225)		
COVID-19 hospitalization (significant)		IVW	5	0.184	0.016	1.202 (1.035-1.395)	0.271	
		MR Egger	5	0.288	0.778	1.334 (0.213-8.364)	0.162	0.918
		Weighted median	5	0.127	0.168	1.135 (0.948-1.360)		

OR, Odds Radio; IVs, Instrumental variables.

With the same approach, we predicted the association between genetically predicted GBM and COVID-19 risk. All MR methods did not indicate an association between genetically predicted GBM and SARS-CoV-2 infection, COVID-19 hospitalization, or severity. MR, heterogeneity, pleiotropy, and sensitivity analyses of all methods associated with genetically predicted GBM and COVID-19 risk are summarized in **Table 2**.

**Table 2:** Association of GBM genetic IVs with COVID-19 GWAS.

Outcome	Exposure	Method	Number of snps	Beta	P	OR (95%CI)	P for heterogeneity test	P for MR-Egger intercept
GBM	SARS-CoV-2 infection	IVW	7	-0.017	0.213	0.983 (0.958-1.047)	0.543	
		MR Egger	7	0.020	0.591	1.020 (0.954-1.050)	0.599	0.299
		Weighted median	7	-0.018	0.269	0.982 (0.952-0.987)		
	COVID-19 severity	IVW	7	-0.045	0.370	0.956 (0.867-1.186)	0.246	
		MR Egger	7	0.073	0.612	1.075 (0.826-1.295)	0.243	0.390
		Weighted median	7	-0.005	0.922	0.995 (0.892-1.068)		
	COVID-19 hospitalization	IVW	7	-0.044	0.171	0.957 (0.899-1.066)	0.745	
		MR Egger	7	0.001	0.988	1.001 (0.851-1.111)	0.677	0.581
		Weighted median	7	-0.057	0.138	0.954 (0.876-1.078)		

OR, Odds Radio; IVs, Instrumental variables.

## 4 Discussion

According to epidemiological studies, cancer is an independent adverse prognostic factor for COVID-19 (3). Due to a higher incidence of GBM in the elderly population, frequent hospitalizations, and treatment-related immunosuppression, COVID-19 is a pandemic that affects many patients with GBM (35, 36). The treatment of patients with high-grade gliomas has already been recommended by several expert groups (6, 37). Some preliminary cross-sectional studies have also supported the hypothesis that patients with GBM are biologically vulnerable to COVID-19 (38, 39). Methodological biases and unmeasured confounders prevented the causality of the association from being established. We investigated the causal relationship between GBM and COVID-19 susceptibility, hospitalization, and severity using public GWAS data.

The results of our MR analyses showed that genetically predicted COVID-19 hospitalization risk significantly increased the risk of GBM in the European population (OR = 1.202, 95% CI = 1.035–1.395, p = 0.016). The following mechanism might explain our results. As a receptor for human Coronavirus-229E (40), Alanyl aminopeptidase (ANPEP) plays an important role in the entry of SARS-CoV-2 into cells (41, 42). Based on its co-expression with ACE2, glutamyl aminopeptidase (ENPEP) has been identified as a candidate co-receptor for SARS-CoV-2 (42, 43). COVID-19 infection results in increased distribution of ENPEP and ANPEP in endothelial cells of the blood-brain barrier, providing the place for SARS-CoV-2 cell entry into the brain. Six receptors were analyzed for survival in GBM cells in a study, and it was found that ANPEP and ENPEP have a beneficial effect on survival. This increases the susceptibility of SARS-CoV-2 to GBM (44).

Healthy human lungs contain large amounts of 27-hydroxycholesterol (27-OHC) produced by macrophages in the alveoli (45). 27-OHC prevents the virus’s lipid envelope from fusing with the host cell membrane, making it difficult for the virus to enter the cell (46). Concentrations of 27-OHC will increase during the presence of COVID-19 infection in lung tissue and blood (47). At the same time, 27-OHC can also promote the growth of glioblastoma tumor cells *in vitro* by stimulating cell division, promoting cell migration and invasion (48). High levels of oxysterols found in glioblastoma tumors isolated from patients were associated with a poorer prognosis. Moreover, the findings confirmed that COVID-19 promotes the malignant behavior of GBM cells. Additionally, there is a possibility that COVID-19-associated coagulopathy could affect long-term tumor behavior and disease progression in GBM in a manner that has not yet been recognized.

According to our MR analyses, genetically predicted GBM risk is not associated with COVID-19 susceptibility, hospitalization, or severity in the European population. This differs from the findings of several preliminary cross-sectional studies (5, 49). Examples of possible confounding factors include the following: the severe lymphopenia often associated with disease or treatment (e.g., alkylating agents like nitrosourea and temozolomide), a frequent presence of neurological deficits resulting in a loss of autonomy in daily living activities and an increased risk of thromboembolic events, an increase in infection susceptibility as a result of chronic use of steroids to treat brain edema, and finally comorbidities and frailties associated with aging. Additionally, since most patients with GBM are hospitalized patients, the detection rate of COVID-19 in this situation may be higher than that in the non-hospitalized population. As a result, the COVID-19 incidence can appear to be higher in cancerous populations when the detection rate is confused with the actual incidence.

Our study has significant clinical implications. First, it has not been clear in the past whether COVID-19 increases the risk of GBM in patients without prior malignancies. In this study, we used Mendelian randomization to reveal that COVID−19 hospitalization increases the risk of developing glioblastoma. This suggests that cancer development is one such foreseeable COVID-19 sequelae since chronic inflammation is long-established to be a fertile ground for oncogenesis, especially for hospitalized patient. Better prevention of COVID-19 and possibly better evidence-based treatment of COVID-19 is warranted in these patients. Second, in our study, genetically predicted GBM risk is not associated with COVID-19 susceptibility, hospitalization, or severity in the European population. This will help us better allocate medical resources.

Our study, however, has several limitations. First, the results of other MR methods showed a consistent but nonsignificant direction. The best results would be obtained if all three methods were significant. The IVW approach, however, is statistically significantly more powerful than the other MR approaches, including MR-Egger (50). The requirement for MR approaches to follow a consistent beta direction has also been strengthened by research. We used this requirement in our study as well (51, 52). In addition, although our data did not show that a genetic predisposition to GBM is associated with COVID-19 susceptibility or severity, it is not appropriate for patients to assume that these patients can be treated at the same level as the general population for medical surveillance management. The third issue is that the sample is of mixed European ancestry. More studies should be carried out on other ethnic groups or immigrants to prove that the same relationship exists. Fourth, we don’t know what percentage of COVID-19 patients already have symptoms that may be related to glioblastoma. We are also unable to analyze the period after COVID-19 when risk arises, and the information provided for control groups remains incomplete.

In conclusion, even though our analysis suggests a causal link between genetically increased COVID-19 and increased risk of GBM, further studies are needed to determine the mechanism behind this association. To optimize the allocation of healthcare resources, it is crucial to identify those who are susceptible to SARS-CoV-2 and those who are prone to severe illness (53).

## Data availability statement

Human data used in this study are publicly available. The database of Genotypes and Phenotypes (dbGaP) contains genotype data from the GWAS for the Glioma International Case-Control Consortium Study under accession phs001319.v1.p1. GWAS Data on COVID-19 is available for download from the website: COVID19-hg GWAS meta-analyses round 5 (covid19hg.org).

## Author contributions

SW and JD drafted the article and contributed to its editing and revision. A bioinformatic analysis was conducted by HX, HZ, MQZ, and WY. JX, YP, MGZ, ZX, ZJ, KW, YW, YM and HC was responsible for the collation and analysis of the data. The manuscript was substantively edited by JD, and all authors have approved the final version.
